# Editorial: Conversation analysis and sociological theory

**DOI:** 10.3389/fsoc.2024.1466130

**Published:** 2024-08-12

**Authors:** Melisa Stevanovic, David Inglis, Emmi Koskinen, Anssi Peräkylä

**Affiliations:** ^1^Faculty of Social Sciences, Tampere University, Tampere, Finland; ^2^Faculty of Social Sciences, University of Helsinki, Helsinki, Finland

**Keywords:** conversation analysis, sociological theory, ethnomethodology, social order, social interaction

The relations between Conversation Analysis (CA), sociology, and social theory are complex, often ambiguous, and have sometimes been rather fraught. CA has both institutional and intellectual roots in the discipline of sociology, especially in the United States (Heritage and Stivers, 2013). Institutionally, CA has existed in often uneasy relations with “mainstream” sociology, sometimes being located within designated “sociology” departments, but often existing outside of them, connecting with other disciplines in creative ways. Intellectually speaking, CA has both rebelled against the parent discipline, while also being the inheritor and elaborator of some of its key themes and ideas (Heritage, 2008).

While there might be agreement amongst their practitioners on what CA is, what it does, and what it is meant to achieve, that is not so much the case for the more open and broad terrains of sociology and social theory. Moreover, each of the domains in question has changed in orientation, composition, and academic location since CA first came into existence in the late 1960s. While initially a child of sociology, as CA has matured and extended its substantive and methodological reach, it has become a large intellectual domain in its own right, with inputs from, and relevance for, a host of other disciplines, notably linguistics, anthropology, and psychology. It is now no longer at all clear how CA relates to sociology and social theory, what each side currently does, or what it could bring to the other in the future.

The Research Topic *Conversation Analysis and Sociological Theory* aims at reflecting upon such matters. It seeks to facilitate a productive dialogue between empirical research on interactional practices and different strands of social and sociological theorizing. In the articles of the Research Topic, researchers in social and sociological theory whose work resonates with conversation analysis have sought to advance the frontiers of knowledge on such matters, critiquing and re-evaluating older positions and elaborating new perspectives on core questions about the nature of human interaction.

## Micro-macro linkage

Early sociological dismissals of CA (e.g., Coser, 1975) regarded it as being ill-equipped to deal with issues of real sociological interest. Yet, CA has over its history been connected with one central problem area of sociology—how “smaller” social phenomena can be related to “larger” ones, and vice versa. Linguistic labels put onto this set of problems include “action” and “structure” relations, and “micro” and “macro” types of phenomena. Such matters are inexorably bound up with issues of how to model forms of social power and power-laden social inequalities (Reed and Weinman, 2018). The twin questions posed by such concerns are: how do power-laden macro-level phenomena manifest themselves in micro-level interactional dynamics, and how does macro-level social order emerge out of those micro-level dynamics? CA needs to be supplemented by social theory in these regards, while simultaneously augmenting the range of social theory's purview of such matters (Arundale).

The early phase of CA (Sacks and Schegloff, 1974) was obviously indebted to two overlapping sets of ideas, each of which had its own distinctive approach to such matters. The first source was Garfinkel's (1967) claims about the meaningful nature of individuals' actions, and social reality as ongoing accomplishment by interacting persons—phenomena which could only be understood by analysts examining the categories used by such persons themselves, instead of deploying conventional sociological categories. The second source was Goffman's (1955) understanding of the nature of human interaction as a profoundly moral exercise, involving the construction and perpetuation of “face”. The notion of conversation as operating according to its own distinctive logics that could be discovered by careful attention to the details of naturally occurring interaction took inspiration from Goffman's notion of the “interaction order” as a *sui generis* entity, which was only “loosely coupled” to other “social institutions” (Goffman, 1983). CA both extended the fight against “conventional” sociology inaugurated by Garfinkel, while significantly contributing to the elaboration of the “interaction order” identified by Goffman. CA found conversation to be a highly structured domain that worked through multiple forms of organizing devices, such as turn-taking (Heritage, 2008).

As CA developed, it came to encompass analyses of the more circumscribed forms of talk that happen within the settings of key social institutions, such as courtrooms and medical facilities (Drew and Heritage, 1992). Such studies illustrated in detail how these institutional contexts actually operate. By adding analysis of transcripts of historically occurring linguistic interactions and comparing these with their present-day counterparts, analysts could demonstrate how changing social norms impacted upon the organization of talk in institutions. This focus on the interrelations between concrete, situational immediacies and organizations (Smith and Stirling), when the latter were examined over time, was a significant step toward understanding how macro-level social change operates in relation to conversational dynamics (Clayman and Heritage, 2002). Analyzing journalistic interactions has also been a resource for understanding how actors' conceptions of “wider society” are manifested in mass media and therefore may influence everyday interactions (Rautajoki).

CA and its ethnomethodologically-inflected variants also developed to encompass the multiple dimensions of interaction (Ayaß), including visual ones (Arminen and Heino). Thus, CA today is a more multifaceted, more interdisciplinary, and more capacious entity than ever before. However, CA's tools to investigate “micro” and “macro” phenomena must be constantly overhauled. Garfinkel's work was once thought by some sociologists to shun considerations of macro-level “structures,” but it is now widely accepted that it contains subtle comprehensions of how social inequalities along the lines of gender and ethnicity play into *in situ* interactions (Duck and Rawls, 2023). While Goffman left open precisely how the “couplings” between the “micro” and “macro” operate (Inglis and Thorpe, 2023), much sociological theorizing since then has endeavored to work out such linkages (e.g., Giddens, 1984; Collins, 2005). Already over the last three decades, work has been done to take CA more in the direction of accounting for forms of social power in interaction, such as in terms of gender and sexualities (Kitzinger, 2005). In this Research Topic, social power is discussed in several articles (see e.g., Ekström and Stevanovic; Stevanovic).

Behind the more obvious Garfinkel/Goffman legacy in CA implicitly lay the ideas of earlier thinkers that those notables themselves had drawn upon, such as the phenomenology of Schutz (1962) and the account of the orderliness of social interaction offered by Durkheim (1984). These and other intellectual resources have subsequently been found to offer both conceptual dead-ends and sometimes surprisingly rich resources for further thinking about how micro-level things relate to macro-level ones. Horgan suggests unexpected but creative resonances between CA and the hermeneutically-attuned version of Durkheim proposed by the Yale School of cultural sociology. To deal more effectively with social hierarchies deeply embedded in certain languages, Sidnell and Vũ propose complementing Durkheim with Marx. Ayaß proposes a fusion of CA with the kinds of theorizing of communication genres pioneered in the German-speaking social sciences. Koskinen et al. demonstrate the resonances between social-theoretical accounts of “recognition” and empirical analyses of esteem, respect and love/care in conversational interactions. Meyer considers the resonances between CA and contemporary practice theories—which already drew upon ethnomethodological insights—in order to reconcile analyses that stress either the context-free or context-sensitive nature of interactional practices.

Perhaps the most radical move in the direction of further connecting CA to bodies of social theory is to do so with reference to assemblage theory in general, and to Actor-Network Theory in particular. The latter denies that there are pre-existing “macro” and “micro” levels and phenomena at all. These, and forms of power, are brought into existence by arranging them into networks of heterogeneous human and non-human actants. Such a perspective opens the possibility of studying the agency of non-humans in human/non-human interactions (Muhle). It also suggests new analytical vistas as to what “social context” might mean in relation to situated interactions, and how larger assemblages relate to localized activities, which are themselves construable as types of assemblage (Raudaskoski).

## Bottom-up vs. top-down

CA's radically empirical enterprise presented an alternative to experimentally driven social psychology and so-called “top-down” deductive social theorizing (Haakana et al., 2009). CA operated in a clear “bottom-up” manner, avoiding premature theory construction (Heritage and Clayman, 2010). However, some recent developments in CA (see e.g., Stivers, 2015) have blurred the clear distinction of top-down/bottom-up analysis. For example, coding interactional phenomena for the purposes of quantification has usually been associated with top-down, theory-driven approaches that involve a risk of an epistemological shift from the member-relevant *emic* accounts *toward* the researcher-relevant *etic* categories (e.g., Markee, 2012; see also Pike, 1967). In CA-informed quantitative studies, however, the coding is usually based on careful, empirical analysis of member-relevant aspects of interaction before moving on to the more researcher-relevant coding of interactional events. In the current Research Topic, a similar complication of a simple top-down/bottom-up distinction in CA occurs with respect to social theory. For instance, in the study conducted by Smith and Sterling, the initial plan for a straightforward bottom-up analysis of conversational data was hindered by methodological obstacles, prompting the authors to delve into theory. Similarly, in the research by Ayaß (p. 1), genre analysis, though firmly rooted in CA methodology, “exceeds it conceptually and theoretically.”

The contexts where CA might face methodological hurdles and where social theory can be helpful include studying power imbalances, atypical populations, and subtle breaches in recognition that escape explicit accountability demands. Ekström and Stevanovic transcend the top-down/bottom-up distinction by holding a conceptual separation of power as an *antecedent* of social action, which can be observed in participants' orientations to their own and each other's accountabilities in various fields of action, and power as a *descendent* of interaction that sheds light on the socially constructed nature of reality, all the while keeping in mind the “dialectic of control” (Giddens, 1984, p. 16) where the two forms of power can be intertwined. Koskinen et al. suggest that violations at the “recognition level” of interaction are particularly difficult to raise to explicit reflective metalevel discussion, as this would necessitate the topicalization of social relations in a way that might become costly for the initiator of the discussion. Hence, to be able to also examine these critical issues they complemented the empirical analysis with concepts and tools gained from recognition theory. Smith and Sterling make the claim that when investigating interactions of atypical populations, the interlocutors can fail to be held to account for transgressions of social order, for example when they start to be oriented-to as mentally ill. In this lack of accountability, there is a weakening of the basis for the “next turn proof procedure” (Sacks and Schegloff, 1974), a fundamental methodological tool for CA scholars. The authors propose that, to solve this dilemma, a dialectic approach might be recommended between processes of model construction and bottom-up processes of observation.

Here, we can observe the presence of a dialectical relationship between deductive, top-down theory and inductive, bottom-up empirical analysis, commonly known as *abductive reasoning* or *abduction* (Peirce, 1935, p. 525). Abductive reasoning “seeks a situational fit between observed facts and rules” (Timmermans and Tavory, 2012, p. 171). Abduction involves a dynamic process of reevaluating data in light of theory, fostering theoretical breakthroughs and novel research hypotheses (Timmermans and Tavory, 2012). While this kind of approach may seem unconventional to some CA scholars, it's worth noting that several foundational categories in CA are already employed in the “bottom-up” analyses, including, for example, turn-constructional units, sequential implication, and epistemic status/stance. The authors of this Research Topic make a deliberate effort to articulate the pre-existing categories and theoretical frameworks they utilize.

Despite the explicit commitment to theorizing, the authors in the Research Topic differ from each other with reference to the role that *conceptualizations* should have in the analysis of social interaction. Several authors share Schegloff's (1997) critique toward the “academic and theoretical imperialism” (p. 165) that “gets to stipulate the terms by reference to which the world is to be understood” (p. 167). Arundale stresses the need to refrain from using conceptual typifications in the analysis of social interaction. Meyer extends such criticism also to encompass the shared practices as reified units of sociality. Others, however, promote the opposite view. According to Alasuutari, “we are born to a world that presents itself through self-evident concepts, the built environment and artifacts, practices, conceptions of proper conduct, and identifications with various communities” (p. 7). The importance of conceptualizations is also highlighted by Mühle, who points to the insight of MCA that “participants in interactions must categorize their counterparts in order create expectations of their activities, motives, and characteristics” (p. 4). If participants themselves display their capability for basic sociological theorizing when dealing with others (Housley and Fitzgerald, 2015, p. 4), researchers shouldn't completely ignore the participants' “folk sociology” when trying to account for their own orientations to what is happening.

Another divide between the articles in the Research Topic is related to the previous one: Should social interaction action be understood solely based on the publicly observable behaviors or are such behaviors to be seen as cues based on which the participants may *interpret* each other's actions, motives, and intentions? While some writers argue that “social order is not created through the interpretive acts of actors” (Duck and Rawls, 2023, p. 246; cited in Arundale), others emphasize the interpretive resources that ordinary members use to account for and justify their own and others' conduct. For example, Horgan refers to “culturally structured collective representations” and “structures of meaning” as resources for such interpretations and argues that people interpret everyday interaction as a moral order. Taking a position along this divide has important implications for research. The former view places the participants themselves and the researchers of interaction into an equal position, as high-quality video-recordings can capture the participants' publicly observable behaviors into a high level of detail. The latter view, in contrast, necessitates that the analysis of interaction considers the cultural context and personal histories of the participants that extend the boundaries of every video-recording.

## Linking theory and CA findings

Looking at the contributions to this Research Topic, we can see an array of sociological concepts that the authors argue have been or can be elucidated by CA findings. Some of these concepts reflect classical themes of sociological theorizing, such as “inequality” (Stevanovic), “division of labor” (Sidnell and Vũ) and “social change” (Mlynar and Arminen). Others reflect more contemporary sociological debates: “assemblage” (Raudaskoski), “recognition” (Koskinen et al.), “societal membership” (Rautajoki), “sociological institutionalism” (Alasuutari), “strong programme in cultural sociology” (Horgan, “agency of non-human entities” (Mühle) and “practice theories” (Meyer). Finally, there are contributions that discuss theoretical concepts that have been developed in dialogue with CA, such as “social emergence” (Arundale), “communicative genres” (Ayaß), and “constitution of common-sense world” (Smith and Stirling).

The task in each contribution is similar: to explore the ways in which CA research has, or can, contribute to the understanding of the given sociological concept and to the ways in which the given concept can elucidate CA. Even though such considerations are not common in earlier research, there are some discussions into which the contributions of this Research Topic can be contextualized. In earlier research, there are broadly two kinds of arguments concerning the linkages between sociological theory and interaction analysis: “generic” and “specific.” The “generic” argumentation offers broad proposals regarding the societal significance of interactional practices. The emergence thesis (discussed and criticized by Arundale) is a case in point: in a rather all-encompassing way, it suggests that the macro-sociological structures emerge from regularities in social interaction. The more “specific” arguments single out particular sociological concepts and particular interactional phenomena. Drew and Heritage's (1992) influential work of institutional interaction is a case in point. It located the concept of institution in the structure of particular (mostly work-related) encounters, and specified facets of the organization of interaction such encounters. Drew and Heritage came up with lexical choice, turn design, sequence organization, overall structural organization, professional neutrality and interactional asymmetries as such facets. The sociological concept of institutional action became thus specified in conversation analytical terms.

The general task of searching for new linkages between CA and the concerns of sociological theory is realized in different ways in different contributions to the Research Topic: in some articles, quite specific CA findings are linked to quite specific theoretical concepts, while in other articles, more generic concerns of sociological theory are linked to CA programme on a more general level. Both types of contributions advance our understanding of CA and sociological theory.

Among the most specific arguments regarding the linkages between sociological concepts and CA findings are to be found in the contribution by Sidnell and Vũ. They explore the significance of the key sociological concept “division of labor” (derived from the classical works of Marx and Durkheim) to the understanding of CA findings concerning practices of other-initiated repair. In their data from Vietnamese conversations, there is a division of labor in the maintenance of intersubjectivity through other-initiated repair: the high and low status participants use different practices of repair initiation. Another contribution suggesting a rather specific linkage between sociological concept and interactional practice comes from Raudaskoski. She discusses contemporary theories on “assemblages” (coming from New Materialism and Actor Network Theory) suggesting, among other things, that Goodwin's (2013) findings of *lamination* can elucidate their local accomplishment as well as the “sociocultural passing on of practices and the material tools involved in them” (Raudaskoski, p. 5).

On a more generic end of arguments regarding the linkages between sociological theories and CA, we find the work of Mlynár and Arminen. The concept that they discuss is most general, “social change”—a concern that arguably penetrates most sociological thinking. While the authors present quite specific CA findings, having to do with the openings of landline and mobile phone calls, their argument is more far-reaching: CA studies on interactions in technological and institutional settings can be read as documentation of practices that in many cases have become or will become obsolete. In some sense, *any* practice discovered by CA can be treated as historical material. The authors thus suggest a new, historically sensitive way of reading CA research.

## The contributions to the Research Topic

The Research Topic consists of 15 articles, which we will summarize very briefly below:

Meyer delves into a detailed examination of the uniquely non-metaphysical, situated way in which the terms “practice” and “practicality” have been understood in ethnomethodology. However, according to Meyer, today's CA tends to reify practices and study them as independent, context-free units of sociality, which points to a gap between ethnomethodology and contemporary CA.

Arundale asks how macro-social order emerges from micro-level human interactions, proposing what he calls the Conjoint Co-constituting Model of Communicating. According to this model, macro-level social systems arise from the recurrent emergence of micro-level social order, as individuals engage in observable social practices across various interactions over time.

Mlynár and Arminen explore the concept of social change due to evolving technologies, which—they argue—can be observed in the details of everyday interactions. The article also discusses the temporal aspects of social practices, proposing that CA studies can serve as historical documents that capture their transient nature, particularly as some practices become obsolete with technological advancements.

Mühle examines the boundaries of the social world, showing how people's basic processes of categorizing their interactional partners can sometimes encompass non-human entities. His analysis, however, points to the moment-by-moment sequentially unfolding and non-determinate nature of such categorization processes, which would need to be better captured by social theorists.

Raudaskoski considers the intersection of CA and the material turn in social sciences, examining two strands of socio-materialism—actor-network theory (ANT) and new materialism(s)—and their emphasis on the entangled nature of practices. Raudaskoski proposes that CA can serve the analysis of these entanglements and highlights the usefulness of Charles Goodwin's concept of lamination in this regard.

Rautajoki examines the construction of society through media communication, delving into how societal membership and collective existence are actualised in media practices. Rautajoki analyzes the opening segments of TV discussions, where journalists address the audience and set the stage for the program's topic, simultaneously ascribing multilayered societal memberships and identities to the participants of the program.

Arminen and Heino operationalize Erving Goffman's concept of civil inattention to explore its impact on relational segregation, highlighting the existence of subconscious monitoring that pre-structures the interaction order. The study also indicates that gaze behavior is influenced by the recipient's appearance, which forms a basis for recognition disparity and can hinder the inclusion of stigmatized groups to the civic sphere.

In his article on interaction ritual, Horgan seeks to build a bridge between CA and cultural sociology. By examining accounts of encounters with rude strangers in public space as breaches of civil inattention, Horgan shows how the analysis of these accounts necessitates the investigation of both local interactional practices and wider structures of meaning that that people use as interpretative resources.

Koskinen et al. draw on recognition theory to analyze complex cases of (mis)recognition. The authors show that solidarity can occur at distinct levels of action and recognition, which can however sometimes be incongruent with each other. The article highlights the capacity of CA to bring the abstract ideas of recognition to life and to inform and promote the development of interactionally based social and societal critique.

Stevanovic discusses the concept of accountability in social interactions and its relation to interactional inequality. Drawing on a distinction between accountability as a tool for making sense of interactions and accountability as a means of maintaining social order, she argues that demands for these two forms of accountability are raised in unfair ways when different groups of people account for their problematic interactional experiences.

Smith and Stirling explore how individuals with schizophrenia may experience disruptions in their common-sense world. Drawing from phenomenological psychiatry and ethnomethodology, the authors present a model of five worlds of meaning and experience. The model is used to elucidate how schizophrenic talk may reflect a loss of integration between these domains.

Sidnell and Vũ discuss the division of labor in the maintenance of intersubjectivity, drawing on the case of repair initiation practices in Vietnamese conversations. The authors argue that these efforts are unevenly distributed among the junior and senior participants. The findings challenge the tacit assumptions of equality in CA and highlight the influence of social hierarchy on conversational practices.

Ayaß discusses genre analysis as a way of bridging language and society. Communicative genres are to be seen as consolidated forms of communication that allow participants to rely on reciprocal orientation and offer solutions to communicative problems. Ayaß substantiates this idea by drawing on three families of communicative genres as examples, presenting CA as the method par excellence for their sequential analysis.

Ekström and Stevanovic argue for an expansion of CA to engage with sociological theories of power, examining how power is interactionally negotiated, but also conditioned by social structures and realities that precede interactional encounters. The authors criticize the tendency in CA to conflate structure and action, which limits the analysis of power in informing action formation.

Alasuutari suggests that insights of neoinstitutional scholarship could help CA to extend its scope to macro-sociological questions. In CA of institutional encounters, participants are seen to negotiate social order under special conditions and restrictions, which often lead into ritualistic ceremonial behavior that is detached from the actors' immediate goals. These rituals play a key role in constituting, maintaining, and naturalizing social order.

## Author contributions

MS: Writing – original draft, Writing – review & editing. DI: Writing – original draft, Writing – review & editing. EK: Writing – original draft, Writing – review & editing. AP: Writing – original draft, Writing – review & editing.
